# Comparative study of whole exome sequencing-based copy number variation detection tools

**DOI:** 10.1186/s12859-020-3421-1

**Published:** 2020-03-05

**Authors:** Lanling Zhao, Han Liu, Xiguo Yuan, Kun Gao, Junbo Duan

**Affiliations:** 1 Department of Biomedical Engineering, School of Life Science and Technology, Xi’an Jiaotong University, Xi’an, China; 2 School of Computer Science and Technology, Xidian University, Xi’an, China

**Keywords:** Copy number variants, Next generation sequencing, Whole exome sequencing, Sensitivity, Specificity, Overlapping consistency, Computational costs, Recommendation, Guideline

## Abstract

**Background:**

With the rapid development of whole exome sequencing (WES), an increasing number of tools are being proposed for copy number variation (CNV) detection based on this technique. However, no comprehensive guide is available for the use of these tools in clinical settings, which renders them inapplicable in practice. To resolve this problem, in this study, we evaluated the performances of four WES-based CNV tools, and established a guideline for the recommendation of a suitable tool according to the application requirements.

**Results:**

In this study, first, we selected four WES-based CNV detection tools: CoNIFER, cn.MOPS, CNVkit and exomeCopy. Then, we evaluated their performances in terms of three aspects: sensitivity and specificity, overlapping consistency and computational costs. From this evaluation, we obtained four main results: (1) The sensitivity increases and subsequently stabilizes as the coverage or CNV size increases, while the specificity decreases. (2) CoNIFER performs better for CNV insertions than for CNV deletions, while the remaining tools exhibit the opposite trend. (3) CoNIFER, cn.MOPS and CNVkit realize satisfactory overlapping consistency, which indicates their results are trustworthy. (4) CoNIFER has the best space complexity and cn.MOPS has the best time complexity among these four tools. Finally, we established a guideline for tools’ usage according to these results.

**Conclusion:**

No available tool performs excellently under all conditions; however, some tools perform excellently in some scenarios. Users can obtain a CNV tool recommendation from our paper according to the targeted CNV size, the CNV type or computational costs of their projects, as presented in Table 1, which is helpful even for users with limited knowledge of computer science.

## Background

Copy number variation (CNV) is a phenomenon that is caused by genomic rearrangement, and the CNV length typically exceeds 1 kilobase (kb) [1]. In medicine, the frequency of CNVs is 12% [2]; hence, it is an important component of gene variations and plays an important role in generating the necessary variation of population and of disease phenotypes [3, 4]. Since the CNV is of substantial medical significance, it has become a hotspot in current medical research, and many accurate CNV identification methods have been proposed in the past two decades. At present, there are many types of CNV recognition methods based on various gene-sequencing methods. Among them are three typical methods: the fluorescence in situ hybridization (FISH), the high-throughput sequencing (HTS) and the array-based comparative genomic hybridization (aCGH) [5]. FISH has a relatively low resolution, and only large repeats can be detected [6]. aCGH only has a high genomic resolution for large CNVs [7]. HTS has a high genomic resolution and can detect not only repetitions but also other types of structural variations in the genome [6]; hence, HTS has become the most popular method for gene sequencing in CNV detection. With the in-depth study of HTS, two branches of research have been developed—the whole genome sequencing (WGS) and the whole exome sequencing (WES). WGS is the laboratory process of determining most DNA base pairs across the 46 chromosomes of an individual’s genome [8], and WES is a process of sequencing all protein-coding regions of genes in a genome [9]. Compared with WGS, WES was proposed later; however, it is more popular in clinical diagnostics and academic research, which due to two advantages of WES: (1) WES is an effective technique for the study of rare Mendelian and common polygenic diseases, such as Alzheimer’s disease; (2) WES is cheaper than WGS. Thus, the CNV detection based on WES has become a research hotspot, and increasingly many have been conducted on methods for detecting CNVs from genes with WES data. However, although increasingly many CNV detection tools are becoming available, there is still no recommended reference for WES-based CNV detection tools in medical applications, which hinders the use of CNV detection tools in practice.

To resolve this problem, in our study, firstly, we chose four tools, which are usually appearing in the literature on CNV tool evaluation and are based on the read depth approach. Then, we comprehensively evaluated their performances in terms of the following three aspects: (1) the sensitivity and specificity of CNV detection for various coverages, CNV sizes and CNV types; (2) the overlapping consistency; (3) the computational cost. Finally, by comparing the performances of these tools, our study not only identifies the limitations and advantages of each CNV tool but also provides a recommended reference on CNV tools according to various requirements, which will facilitate researchers in the selection of suitable tools for their projects.

## Conclusions

In this study, first, we selected four WES-based CNV detection tools: CoNIFER, cn.MOPS, CNVkit and exomeCopy. Then, we comprehensively evaluated and compared the performances of four selected CNV tools. Finally, by analyzing the experimental results, we recommend suitable CNV tools according to the application requirements, which are listed in Table 1. According to this table, we can divide the requirements into two categories: (1) The accuracy is the primary comparison criterion. (2) The accuracy is not the primary comparison criterion. In the first category, if the target CNV sizes are small, the recommendation is CNVkit; otherwise, the recommendation is cn.MOPS. If the CNV insertions are more frequent than the CNV deletions, the recommendation is CoNIFER; otherwise, CNVkit is a satisfactory choice. However, in practice, the information of the target CNVs is typically unknown, and the recommendation of our study is to use CoNIFER and cn.MOPS together. In the other category, if the researcher must deal with large WES data rapidly and desires a typical sensitivity, the recommendation is cn.MOPS, whereas if the researcher wants to detect CNVs on any computer, including a low-configuration computer, the recommendation is CoNIFER. Thus, in practice, we can recommend a proper CNV tool to researchers according to their requirements, which can maximize the accuracy of the test result. For instance, according to Rohrback’s study [10], the size of a CNV that is in a neuron of the human brain is typically between 2 Mb and 10 Mb, and to obtain an accurate result for its CNV detection, the recommended CNV tool is cn.MOPS.

**Table 1:** The recommended tool for different requirements

Requirements		Recommendation
Accuracy first	CNV size is small(< 100 kb)	CNVkit
	CNV size is large	cn.MOPS
	More insertion	CoNIFER
	More deletion	CNVkit
	No prior knowledge	cn.MOPS+CoNIFER
Others	Speed first	cn.MOPS
	Low- memory	CoNIFER

## Method

### Data sets

In this study, we used simulated WES data and real WES data to evaluate the performances of CNV tools. For simulated data, the CNV size, the CNV number and the coverage are available to the researchers, while those of real data are unknown.

For the simulated data, we used hg38 as the reference genome, which can be downloaded from The National Center for Biotechnology Information (NCBI), https://www.ncbi.nlm.nih.gov/. Then, we simulated the test genome by using the reference genome, and in this process, three assumptions were made: (1) The CNV size exceeds 1 kb. (2) The coverage is 100X. (3) The length of the reads is 50 bp. After obtaining the test genome and the reference genome, we used bowtie2 and samtools with their default parameters to obtain the aligned BAM file, which can be used by CNV tools directly.

For the real data, we selected ten real samples from the exome example of CNVkit, which can be downloaded from GitHub, https://github.com/etal/cnvkit-examples/tree/master/exome. Then, to enable the use of these data with other selected CNV tools, we converted these samples from cnn files to GRange objects and S4 objects; in addition, we created files in which the reads per kilobase per million mapped reads (RPKM) are recorded. After these steps, we can obtain real data that can be used with these CNV tools directly.

### Tools’ comparisons

To facilitate researchers in the selection of suitable WES-based CNV tools according to their requirements, we selected representative tools and evaluated their performances in various cases. In this process, one of tool selection criteria is that they are based on the read depth approach for the detection of CNVs, which assumes that the variation in the read depth is unbiased, random and normally distributed and that the deviation from the background document may signify the presence of a CNV [11–13]. The other selection criterion is the availability of the tools.

With these criteria, first, we identified candidate tools from previous studies on the evaluation of CNV detection tools [14–16], such as CoNIFER [17], exomeCopy [18], ExCopyDepth [14], CNVkit [19], cn.MOPS [20] and so on. Then, we attempted to download these candidate tools. In the process, because the resources of some tools were unavailable, we screened out several of the CNV tools, such as ExCopyDepth. Finally, we selected four tools as the final tools by comparing the weekly downloads of candidate tools, and information on them is presented in Table 2.

**Table 2:** Selected representative CNV calling methods

Tools	Language	Input format	Methodology	Installation tutorial	Reference
CoNIFER	python	BAM/RPKM、bed、RPKM	Principal Component Analysis	http://conifer.sourceforge.net/index.html	Krumm. et al.(2012) [17]
exomeCopy	R	BAM、bed、Grange	A hidden Markov model which uses positional covariates	http://www.bioconductor.org/packages/release/bioc/html/exomeCopy.html	Love. et al.(2011) [18]
CNVkit	python	BAM、bed、cnn	Principal Component Analysis	https://cnvkit.readthedocs.io/en/stable/	Eric. et al.(2016) [19]
cn.MOPS	R	BAM、bed、Grange	Bayesian approach	http://www.bioinf.jku.at/software/cnmops/cnmops.html	Klambauer. et al.(2012) [20]

According to Table 2, our selected CNV tools are CoNIFER, exomeCopy, CNVkit and cn.MOPS. Among them, CoNIFER uses singular value decomposition (SVD) to eliminate capture biases between sample batches and to detect CNVs in exome data [17]. exomeCopy uses the hidden Markov model (HMM) to detect CNVs in exome data [18]. CNVkit uses both targeted reads and nonspecific captured off-target reads to infer the copy number evenly across the genome for the detection of CNVs in exome data [19]. cn.MOPS uses Bayesian inference to detect CNVs in the exome data [20]. Tools differ in terms of their methods, which typically causes their performances to differ among cases. In practice, while using these CNV tools, we need to adjust their parameters to realize their optimal performances in various cases. However, this workload is heavy, and the simulation of all cases is unrealistic. Therefore, we chose a compromise in our study and adjusted parameters of each of the tools by evaluating their comprehensive performances for simulated data for which the CNVs’ sizes range from 1 kb to 10 Mb randomly. Our recommended parameters for these CNV tools are as follows: For CoNIFER, we set *svd* to 1. For exomeCopy, we set *relto* to 0.01, *goto.cnv* to 0.0001 and *goto.normal* to 0.01. For CNVkit, we set *target-avg-size* to 300 bp. For cn.MOPS, we set *priorImpact* to 20, *min.width* to 9, *upperThreshold* to 0.4 and *lowerThreshold* to the default. All the experimental results of parameter adjustment are shown in additional file 1.

### Comparison criteria

To more comprehensively assess the tools, we evaluated these tools mainly in terms of the sensitivity and specificity, overlapping consistency and computational costs. The comparison criteria are described in detail as follows.

### Sensitivity and specificity

In medicine, sensitivity and specificity are two statistical measures that are widely used to evaluate the performance of a binary classification test [21]. Sensitivity is the statistic that measures the proportion of positive results that are correctly identified as such, and specificity is the statistic that measures the proportion of negative results that are correctly identified as such [22]. As in other medical dichotomy experiments, in our study, we used sensitivity and specificity as criteria for the performance evaluation of these CNV tools. In the process of calculating the sensitivity and specificity, we used the exon as the minimum unit, and calculation formulas for the sensitivity and specificity are presented as follows. The sensitivity is calculated via eq. (1) and is defined as the true positive rate (TPR). The specificity is calculated via eq. (2) and is defined as the true negative rate (TNR).

1 $$ TPR=\frac{TP}{P}=\frac{TP}{TP+ FN} $$

2 $$ TNR=\frac{TN}{N}=\frac{TN}{TN+ FP} $$

In these equations, there are six values: positive (P), negative (N), true positive (TP), true negative (TN), false positive (FP) and false negative (FN). They are defined in Table 3. From this table, we can obtain not only the definitions of these values but also the relationships among them, such as TP plus FN equals P and TN plus FP equals N.

**Table 3:** The definition of six values in sensitivity and specificity

Values	Definition
P (Positive)	Number of exons which are within the real CNV
N (Negative)	Number of exons which are out of the real CNV
TP (True Positive)	Number of exons which are within both the detected CNV and the real CNV
TN (True Negative)	Number of exons which are out of both the detected CNV and the real CNV
FP (False Positive)	Number of exons which are within the detected CNV and out of the real CNV
FN (False Negative)	Number of exons which are out of the detected CNV and within the real CNV

### Overlapping consistency

The overlapping consistency is a comparison method that is typically used in medicine. In our study, since ground-truth information for the real data is unavailable, we abandoned the strategy of evaluating the performances of these CNV tools by comparing their sensitivity and specificity values under various conditions; instead, we used the Venn diagram [23–25] to evaluate their performances on real data. During this process, since the differences in the sample types may affect the results of the overlap test, we conducted this test for not only real data but also simulated data, and used the result for simulated data as the reference for the real data. To facilitate the evaluation, we quantified the consistency among these four tools. Here, we introduce the overlap rate as the quantitative value, which is defined as the ratio between N_(overlap) and N, where N is the total number of exons that are detected by a CNV tool and N_(overlap) is the number that overlapped with those that were detected by the other tools. For example, from Fig. 4a, for cn.MOPS, *N* = 6405, and N_(overlap) = N-58; hence, the overlap rate is 99%. This process aims at determining the consistency of these tools’ results. If the overlap rates detected by these tools are high, the results of these tools have high CNV consistency and are trustworthy.

### Computational costs

Computational costs are typically used to evaluate the performance of an algorithm in computer science. To make our assessment comprehensive, we assessed these CNV tools in terms of not only statistical characteristics but also computational costs. In the evaluation, computational costs include the time complexity and the space complexity. The time complexity refers to the computational effort that is required to execute the algorithm, which can be represented by the product of the central processing unit (CPU) utilization and the average running time. The space complexity is a measure of the size of the storage space that is required by the algorithm, which can be represented by the memory occupancy.

## Results

### Sensitivity and specificity

In our study, we used the sensitivity and the specificity to evaluate the performances of these selected tools. In this process, since the coverage, CNV size and CNV type of the WES data may influence the performances of tools, we simulated three types of data and studied the changes in the CNV tools’ performances with respect to these three factors. The results are presented as follows.

### Coverage

**Fig. 1:**
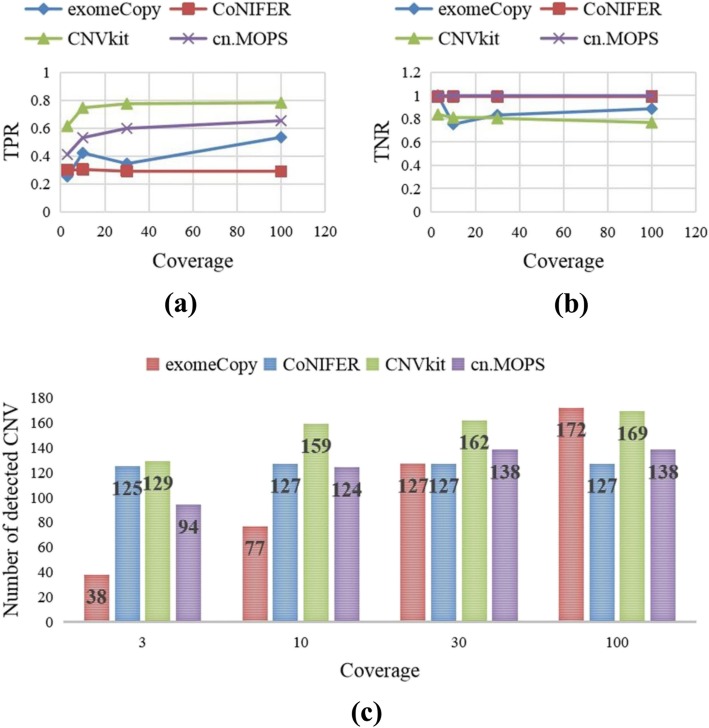
The changes of tools’ performances with respect to the coverage. Fig **a** and **b** describe the changes of these tools’ sensitivities (TPRs) and specificities (TNRs) with respect to the coverage, and Fig **c** describes the numbers of detected CNVs in different coverages for these tools.

To evaluate the impact of the coverage on the CNV detection performances of these tools, we considered a series of WES datasets with coverages of 3X, 10X, 30X and 100X for which the probability of insertions is equal to the probability of deletions. Then, we used the selected tools to detect CNVs from these data. The results are presented in Fig. 1. Figure 1a and b presents the changes of these tools’ sensitivities (TPRs) and specificities (TNRs) with respect to the coverage, and Fig. 1c presents the numbers of detected CNVs by these tools with various coverages.

From Fig. 1, we obtain three main conclusions: First, the sensitivity (TPR) increases rapidly and subsequently stabilizes with the increase of the data’s coverage, which may be caused by the ceiling effect. Second, the specificity (TNR) decreases overall with the increase of the sensitivity. Finally, the number of detected CNVs of every tool increases initially and subsequently remains unchanged with the increase of the data’s coverage. According to these results, the coverage of 100X is sufficient in practice, for which the sensitivities and specificities of these tools are satisfactory, and the computational burden is much lower than that for the data with higher coverage.

### CNV size

**Fig. 2:**
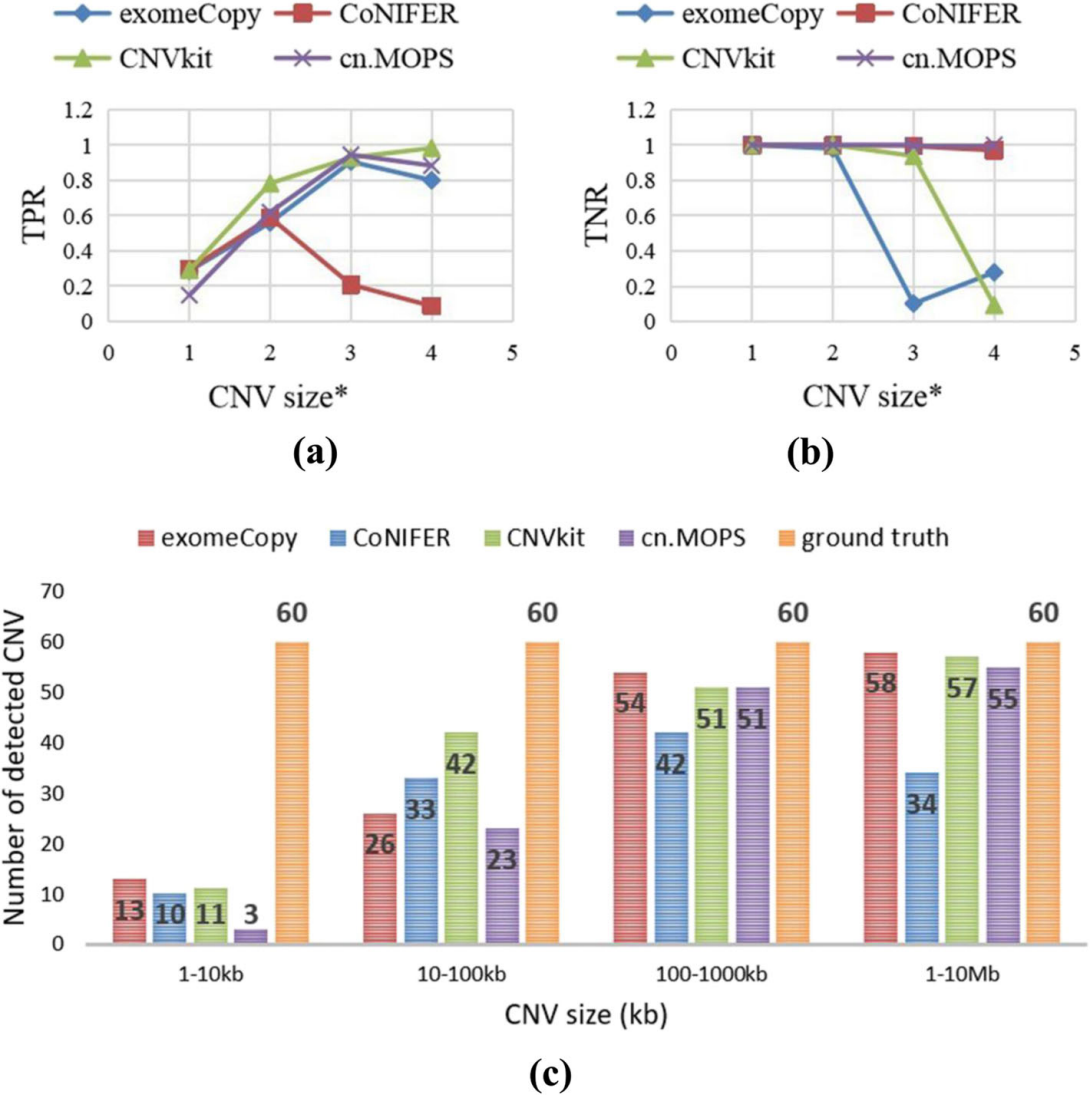
The changes of tools’ performances with respect to the CNV size. Fig **a** and **b** show the changes of these tools’ sensitivities (TPRs) and specificities (TNRs) with respect to the CNV size, and Fig **c** shows the numbers of detected CNVs in different CNV sizes for these tools.

To evaluate the influence of the CNV size on the CNV detection performance, we simulated a series of datasets as input, for which the CNV sizes are distributed in 1 kb–10 kb, 10 kb–100 kb, 100 kb-1 Mb and 1 Mb–10 Mb while the coverage is 100X and each CNV type (deletion and insertion) occurs with equal frequency among them. Then, we used the selected tools to detect CNVs from these datasets. The results are presented in Fig. 2. Figure 2a and b show the changes of these tools’ sensitivities (TPRs) and specificities (TNRs) with respect to the CNV size, and Fig. 2c shows the numbers of detected CNVs of various CNV sizes for these tools. For the abscissa axis of Fig. 2a and b, the CNV size* is a value that is computed from the CNV size by dividing the CNV size by 1000, calculating the base 10 logarithm, and rounding up the value. For example, when the CNV size is 111 kb, the CNV size* is 3, and when the CNV size is 9 Mb, the CNV size* is 4.

From Fig. 2, we draw two main conclusions: First, for all these tools, the sensitivity increases initially and subsequently remains unchanged or decreases slightly with the increase of the CNV size, while the specificity decreases as the sensitivity increases, and the number of detected CNVs increases as the CNV size increases. Second, the performances of these tools change with the CNV size, and the recommended tools differ among the cases. For example, when the targeted CNV size is between 1 kb and 100 kb, CNVkit comprehensively outperforms other tools in terms of the sensitivity and specificity, whereas when the targeted CNV size is between 100 kb and 10 Mb, cn.MOPS performs best comprehensively in terms of the sensitivity and specificity.

After we obtained the sensitivities and specificities of these tools for various CNV sizes, since the targeted CNVs may be unknown, we calculated the global sensitivities and specificities of these tools by averaging their sensitivities and specificities over various CNV sizes. The results are presented in Table 4. According to the information in this table, cn.MOPS is a suitable choice for unknown research as its specificity and sensitivity are satisfactory comprehensively.

**Table 4:** The global sensitivity and the global specificity of four CNV tools

CNV detection tool	ExomeCopy	CoNIFER	CNVkit	cn.MOPS
Sensitivity (TPR)	0.64	0.29	0.75	0.65
Specificity (TNR)	0.60	0.99	0.76	1.00

### CNV type

**Fig. 3:**
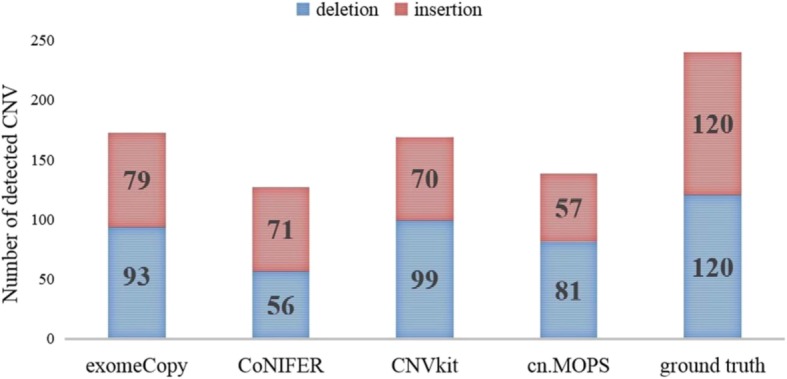
The number of detected CNV for different CNV type.

To determine whether the CNV type influences the CNV detection or not, we simulated a series of datasets, of which the coverage is 100X, the CNV size is random and the CNV types occur with equal frequency. Then, we used the selected tools to detect CNVs and counted the number of detected CNVs of each type. The results are presented in Fig. 3.

From Fig. 3, we conclude the following: First, all these tools can detect not only CNV insertions but also CNV deletions. Second, all tools except CoNIFER perform better for CNV deletions than for CNV insertions. Third, although CoNIFER performs better for insertions than for deletions, it may not perform the best among all these tools for insertions, of which the performance also depends on the distribution of the CNV size.

### Overlapping consistency

In our study, to evaluate the consistency of these CNV tools, we conducted overlap tests on the simulated data and real data.

**Fig. 4:**
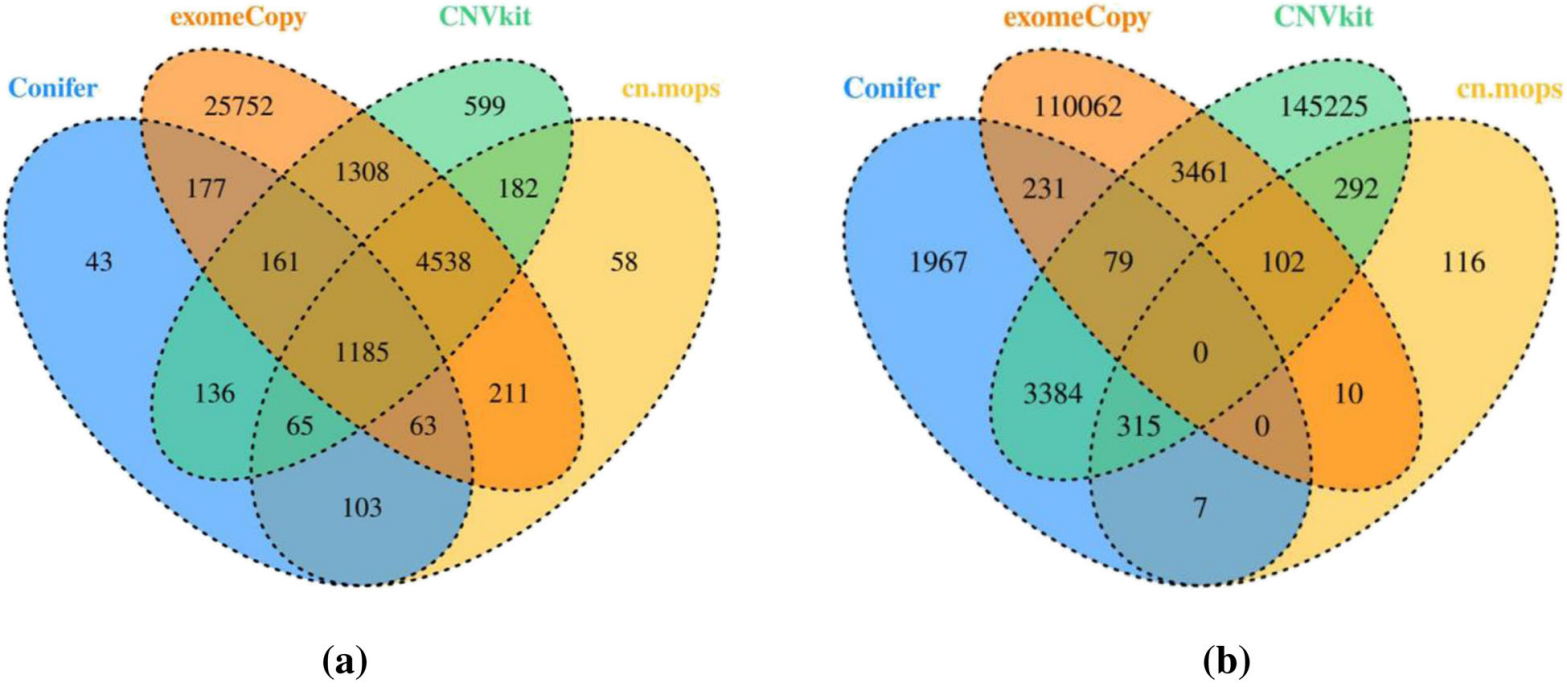
The overlapping consistency results. Fig **a** and **b** show the simulated data and real data.

For the simulated data, first, we simulated a series of datasets, of which the coverage is 100X and the CNV size and type are random. Then, we used the selected four tools to detect CNVs. Finally, we drew a Venn diagram of the detection results, which is shown in Fig. 4a.

For the real data, first, we downloaded exome examples from CNVkit and used them as the original data. Then, we converted the original data (in cnn format) into the formats that are required by the other three CNV tools: RPKM format for CoNIFER, GRange format for exomeCopy and S4 for cn.MOPS. Finally, we detected CNVs and drew a Venn diagram by following the same procedure as for the simulated data. The Venn diagram is presented in Fig. 4b.

With the information in Fig. 4, we calculated the overlap rates (defined in section Comparison criteria) of these four tools to quantify their consistency, which are listed in Table 5.

**Table 5:** The overlap rates of four CNV tools

CNV detection tool	ExomeCopy	CoNIFER	CNVkit	cn.MOPS
simulated data	0.23	0.98	0.93	0.99
real data	0.03	0.67	0.05	0.86

According to Table 5, the overlap rates of CoNIFER, CNVkit and cn.MOPS exceed 90% for the simulated data; hence, they realize satisfactory consistency in the detection of CNVs, and their results are highly trustworthy. In addition, cn.MOPS and CoNIFER also realize satisfactory consistency (86 and 67%) on the detection of CNVs from real data.

However, not all of these tools realize satisfactory consistency. The overlap rate of exomeCopy is always low (23% on simulated data and 3% on real data). To determine the cause of this phenomenon, we reviewed many other studies and found that our result is similar to Tan’s results (22%) [24], according to which the overlap rate of exomeCopy is associated with its algorithms.

**Fig. 5:**
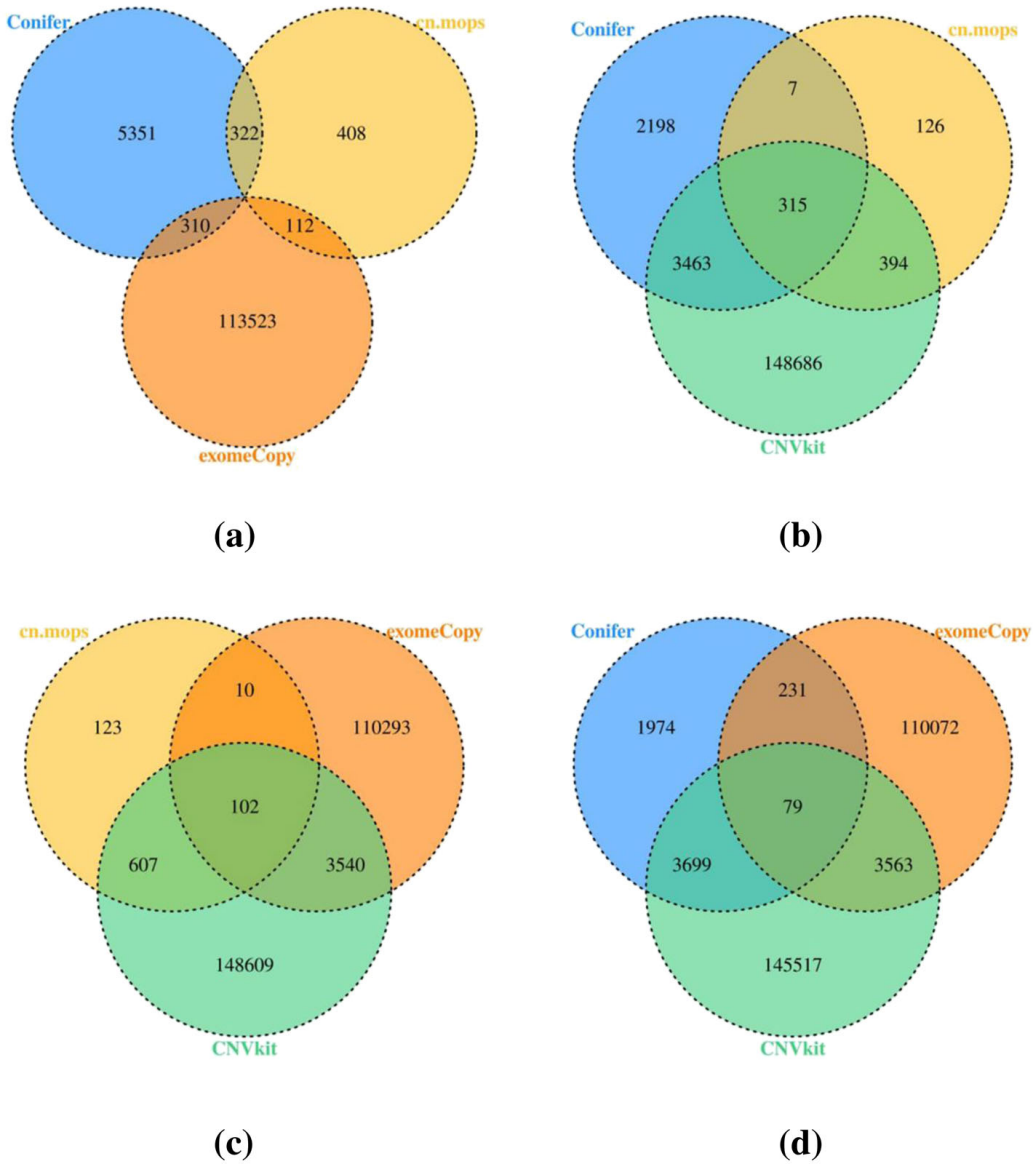
Venn diagrams of three CNV tools. Fig **a** is for CoNIFER, cn.MOPS and exomeCopy, Fig **b** is for CoNIFER, cn.MOPS and CNVkit, Fig **c** is for CNVkit, cn.MOPS and exomeCopy, and Fig **d** is for CoNIFER, CNVkit and exomeCopy.

In addition, we found that the tools’ overlap rates for simulated data are higher than those for real data in our test. To determine what led to this phenomenon, we made the Venn diagrams of three of four tools, which were selected randomly. The results are presented in Fig. 5.

According to Fig. 5, all the combinations of three of these four tools have common exons except the combination of exomeCopy, cn.MOPS and CoNIFER, which is because the number of detected exons by cn.MOPS is too small relative to those by other tools. However, from the detection results on the simulated data, cn.MOPS outperformed most of the tools in terms of global sensitivity and specificity, which is not in line with the result of overlapping consistency. Based on the results from the simulated data, we think the underlying causes of this phenomenon may be that the CNV sizes of the samples don’t focus on 10 kb to1 Mb and the number of CNV insertions exceeds the number of CNV deletions, which may cause the numbers of false detections for exomeCopy and CNVkit to be far larger than those for cn.MOPS and CoNIFER.

### Computational costs

To assess these CNV tools comprehensively, we also used the computational cost as an evaluation criterion, which includes the time complexity and the space complexity. The results are presented as follows.

### Time complexity

**Fig. 6:**
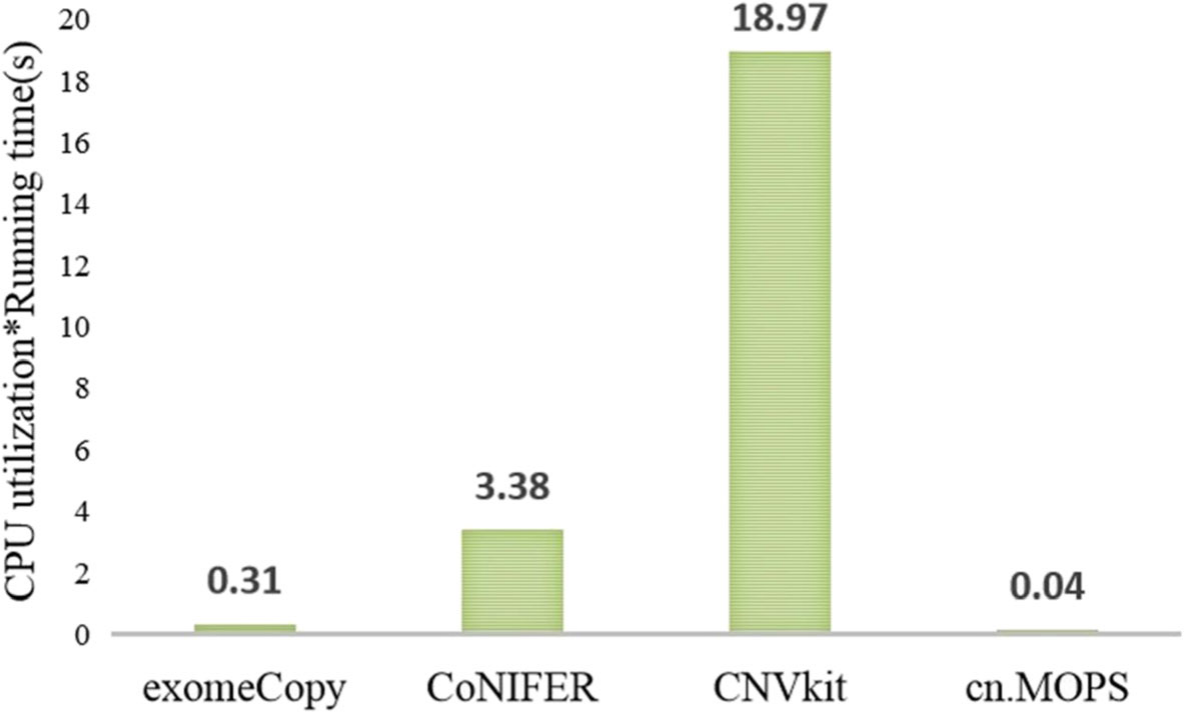
The time complexities of exomeCopy, CoNIFER, CNVkit and cn.MOPS.

In our study, to determine the time complexities of these tools, we simulated a series of datasets as input, of which the coverage is 100X and the size is close to 11.2 MB. Then, since we do not have the detailed algorithm of these tools, we calculated the time complexity of each tool by multiplying the average running time and the CPU utilization. The results are presented in Fig. 6.

According to Fig. 6, cn.MOPS has the lowest time complexity; hence, it will require the minimum time for the same data processing among these tools. CNVkit has the highest time complexity, while it realizes satisfactory sensitivity and specificity.

### Space complexity

**Fig. 7:**
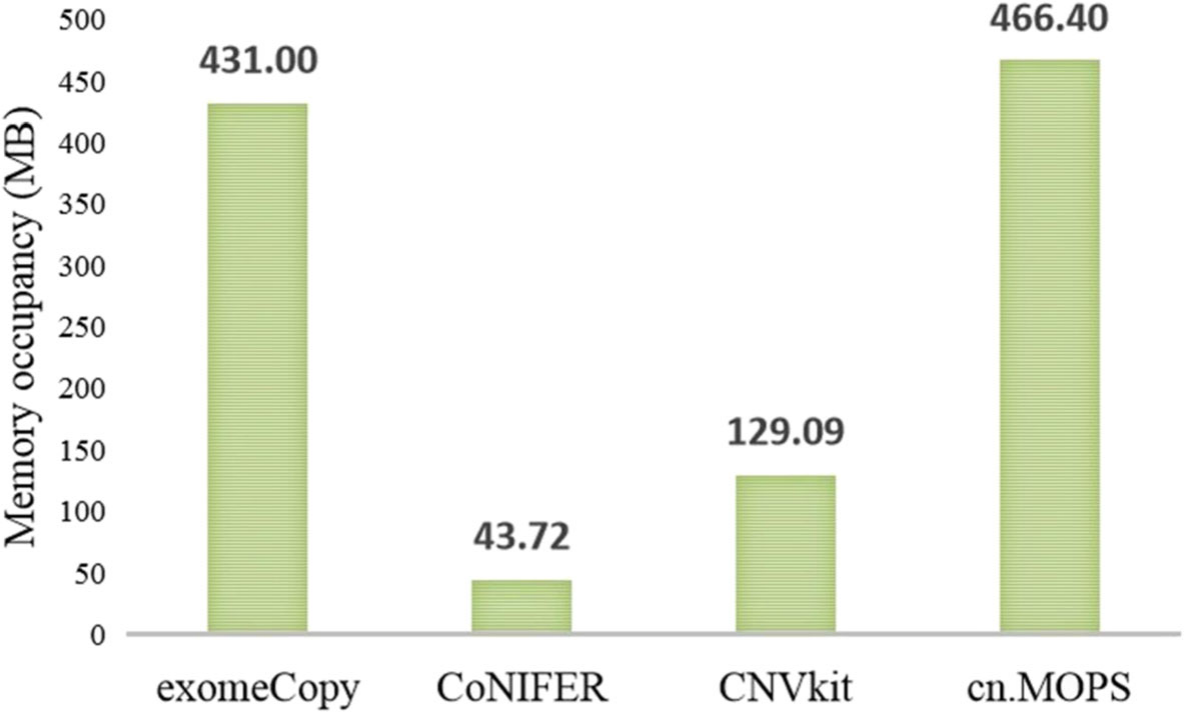
The space complexities of exomeCopy, CoNIFER, CNVkit and cn.MOPS.

To determine whether the CNV tool will affect other programs while it is running, we simulated a series of datasets as input, of which the coverage is 100X and the size is close to 11.2 MB. Then, we used the selected tools to detect CNVs from these datasets and calculated the average memory occupancy as a characterization quantity of the space complexity. The results are presented in Fig. 7.

According to the information in Fig. 7, CoNIFER has the lowest memory occupancy for the same data processing among these tools; hence, it has the minimum requirements for computer hardware. cn.MOPS has the highest memory occupancy among these tools, while it has the lowest time complexity, and this is because the time complexity and the space complexity are mutually constrained.

## Discussion

With the development of CNV detection based on WES, increasingly many tools are being proposed and evaluated. However, no reference guide of CNV tools recommendation is available for various cases. Hence, the application of CNV tools in the clinic is challenging.

To facilitate clinicians and investigators in the selection of suitable CNV tools, we reviewed and compared prominent studies on evaluating CNV tools [23–25], and we identified three main areas for improvement: (1) the evaluation method. Most studies compared CNV tools only in terms of statistical characteristics, such as the sensitivity and specificity, and the influences of the coverage, CNV size and CNV type on tools’ statistical characteristics were not considered. Therefore, in our study, we evaluated the performances of CNV tools in terms of not only statistical characteristics but also computational costs; in addition, we evaluated the influence of CNV characteristics; (2) the selection of CNV tools. In most studies, the latest tools were selected; however, the perfective maintenance stage in the software life cycle and user evaluations were not considered; therefore, in our study, we selected CNV tools according to the frequency of application, which is represented by the download frequency and the number of citations in the literature; (3) the practical guideline. Most studies identified advantages and disadvantages of tools without considering the users’ background. Therefore, it is difficult for users to choose suitable tools for their projects. In our study, we recommend suitable CNV tools according to various conditions, which is helpful for users who have limited knowledge of computer science.

In the study, we have selected four representative WES-based tools and evaluated their performances from three aspects. From results of this study, we obtain information about the performances of these tools in various cases. Then, by comparing their performances, the most suitable tool can be identified in various scenarios, which will facilitate researchers in the selection of proper tools according to their projects. For example, among these tools, CNVkit has the highest sensitivity and a typical specificity for CNVs of which sizes are between 10 kb and 100 kb; hence, CNVkit is the best choice for CNVs with sizes that are in this range.

Based on this study, we can recommend suitable CNV tools to researchers according to their projects, which can promote the clinical application of these tools. However, there are still many areas for improvement in this study: first, in this study, we selected only four WES-based tools as representatives for performance evaluation, and the guideline for CNV detection based on WES can be improved by adding more tools to this comparison. Second, the guideline is mainly for WES data with a coverage of approximately 100X, and the performances of these tools for low coverage data are unknown in various cases. Finally, this study only evaluated the performances of WES-based CNV tools and ignored the comparison between WES-based CNV tools and WGS-based CNV tools.

## Supplementary information

**Additional file 1:** Selection of tool parameters. A text includes all the figures and tables about tool parameters’ selection.

## Data Availability

All data used in this study are available from the corresponding author upon request.

## Abbreviations

aCGH: Array-based comparative genomic hybridization
CNV: Copy number variation
FISH: Fluorescence in situ hybridization
HMM: Hidden Markov model
HTS: High-throughput sequencing
kb: kilobase
Mb: Mbase
MB: MByte
NCBI: National Center for Biotechnology Information
RPKM: Reads per kilobase of exon model per million mapped reads
TNR: True negative rate
TPR: True positive rate
WES: Whole exome sequencing
WGS: Whole genome sequencing
