# Supplementary material for: Comparative study of whole exome sequencing-based copy number variation detection tools
Source: BMC Bioinformatics. 2020 Mar 5;21:97. doi: 10.1186/s12859-020-3421-1 (PMC7059689; doi:10.1186/s12859-020-3421-1)
Supplement: Supplementary file 1 — Additional file 1: Selection of tool parameters. A text includes all the figures and tables about tool parameters’ selection. [file 12859_2020_3421_MOESM1_ESM.pptx]

## Slide 1
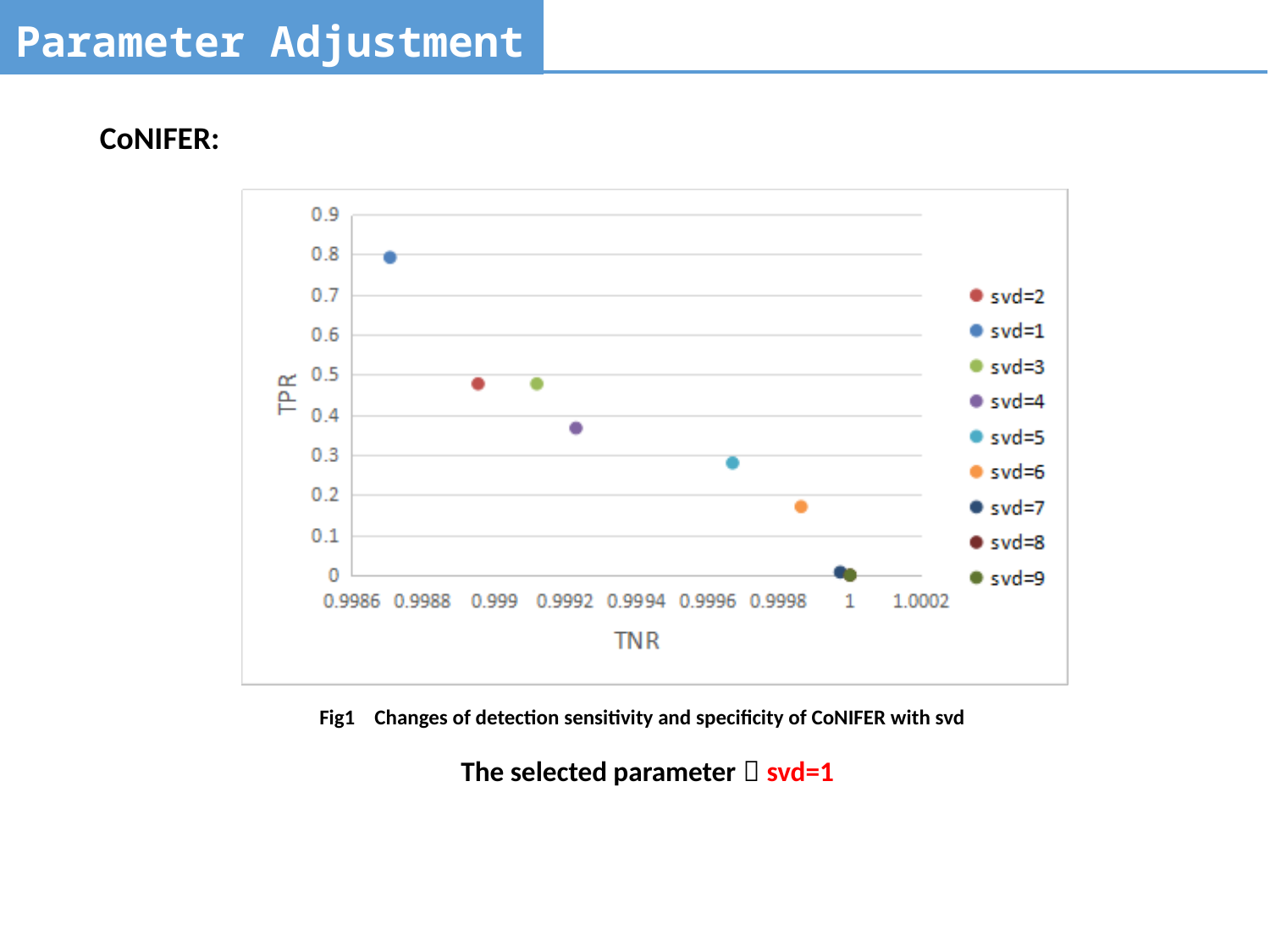

Parameter Adjustment
CoNIFER:
Fig1 Changes of detection sensitivity and specificity of CoNIFER with svd
The selected parameter：svd=1

## Slide 2
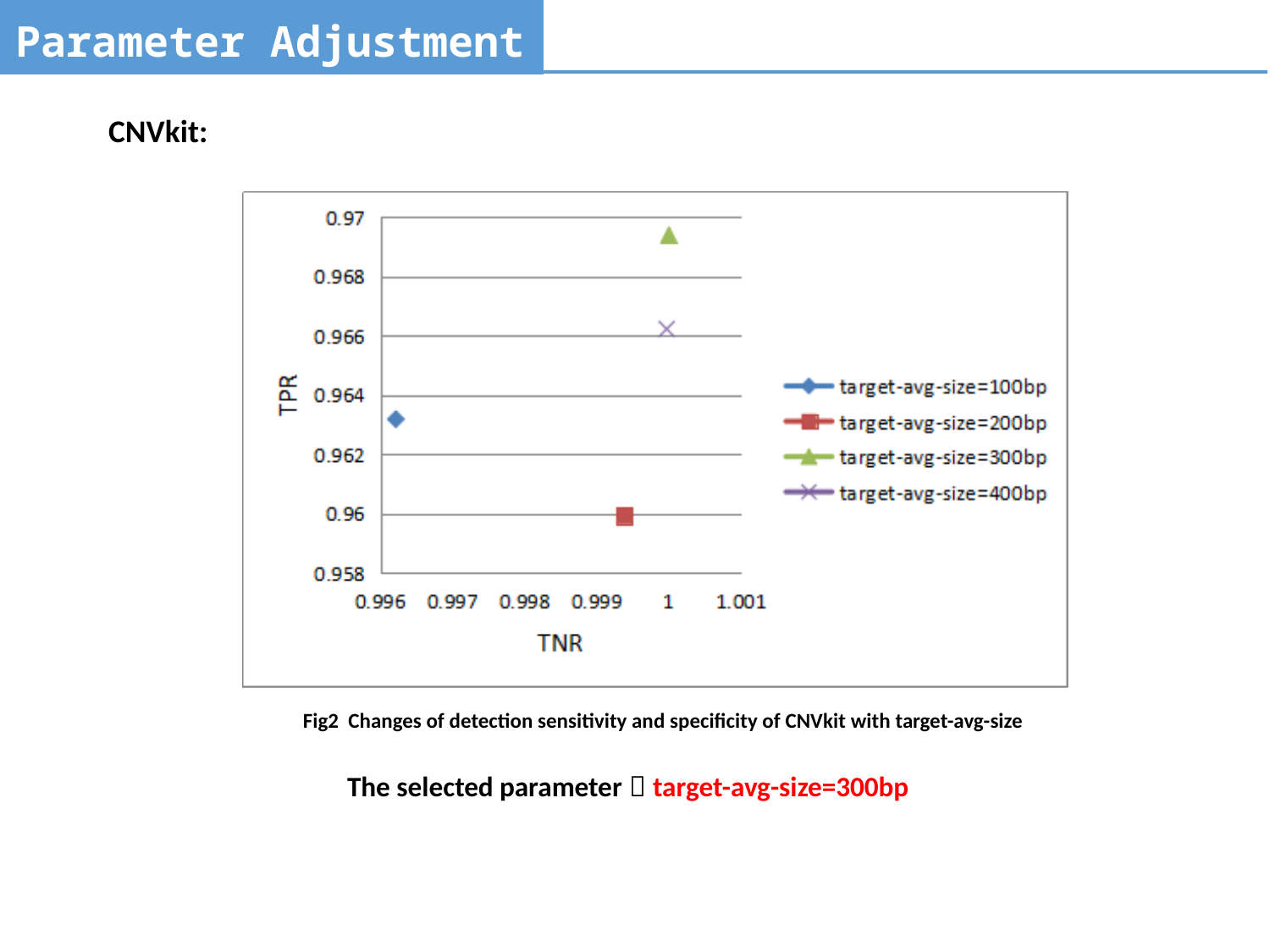

Parameter Adjustment
CNVkit:
Fig2 Changes of detection sensitivity and specificity of CNVkit with target-avg-size
The selected parameter：target-avg-size=300bp

## Slide 3
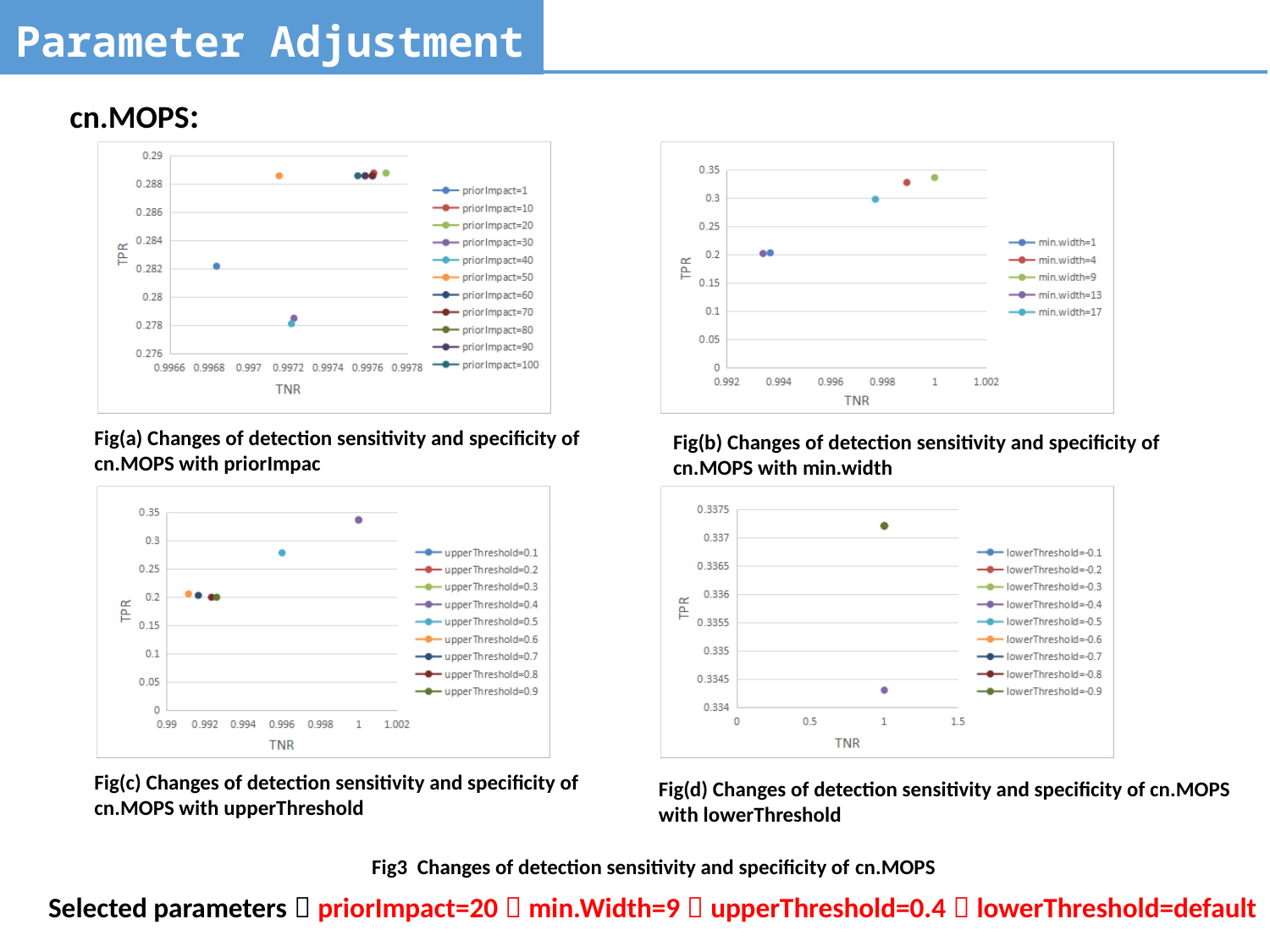

Parameter Adjustment
cn.MOPS:
Fig(a) Changes of detection sensitivity and specificity of cn.MOPS with priorImpac
Fig(b) Changes of detection sensitivity and specificity of cn.MOPS with min.width
Fig(c) Changes of detection sensitivity and specificity of cn.MOPS with upperThreshold
Fig(d) Changes of detection sensitivity and specificity of cn.MOPS with lowerThreshold
Fig3 Changes of detection sensitivity and specificity of cn.MOPS
Selected parameters：priorImpact=20，min.Width=9，upperThreshold=0.4，lowerThreshold=default

## Slide 4
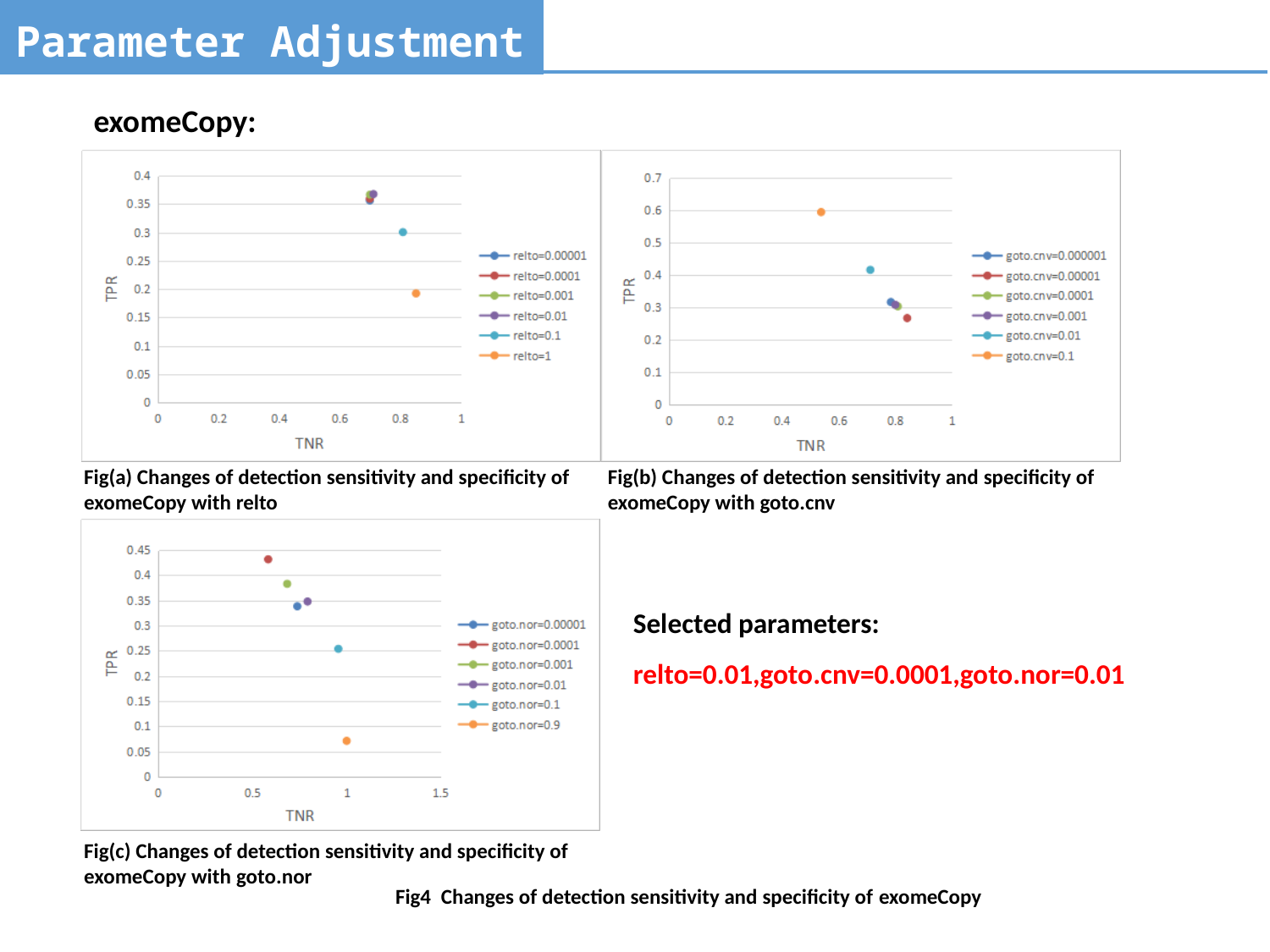

Parameter Adjustment
exomeCopy:
Fig(a) Changes of detection sensitivity and specificity of exomeCopy with relto
Fig(b) Changes of detection sensitivity and specificity of exomeCopy with goto.cnv
Selected parameters:
relto=0.01,goto.cnv=0.0001,goto.nor=0.01
Fig(c) Changes of detection sensitivity and specificity of exomeCopy with goto.nor
Fig4 Changes of detection sensitivity and specificity of exomeCopy

## Slide 5
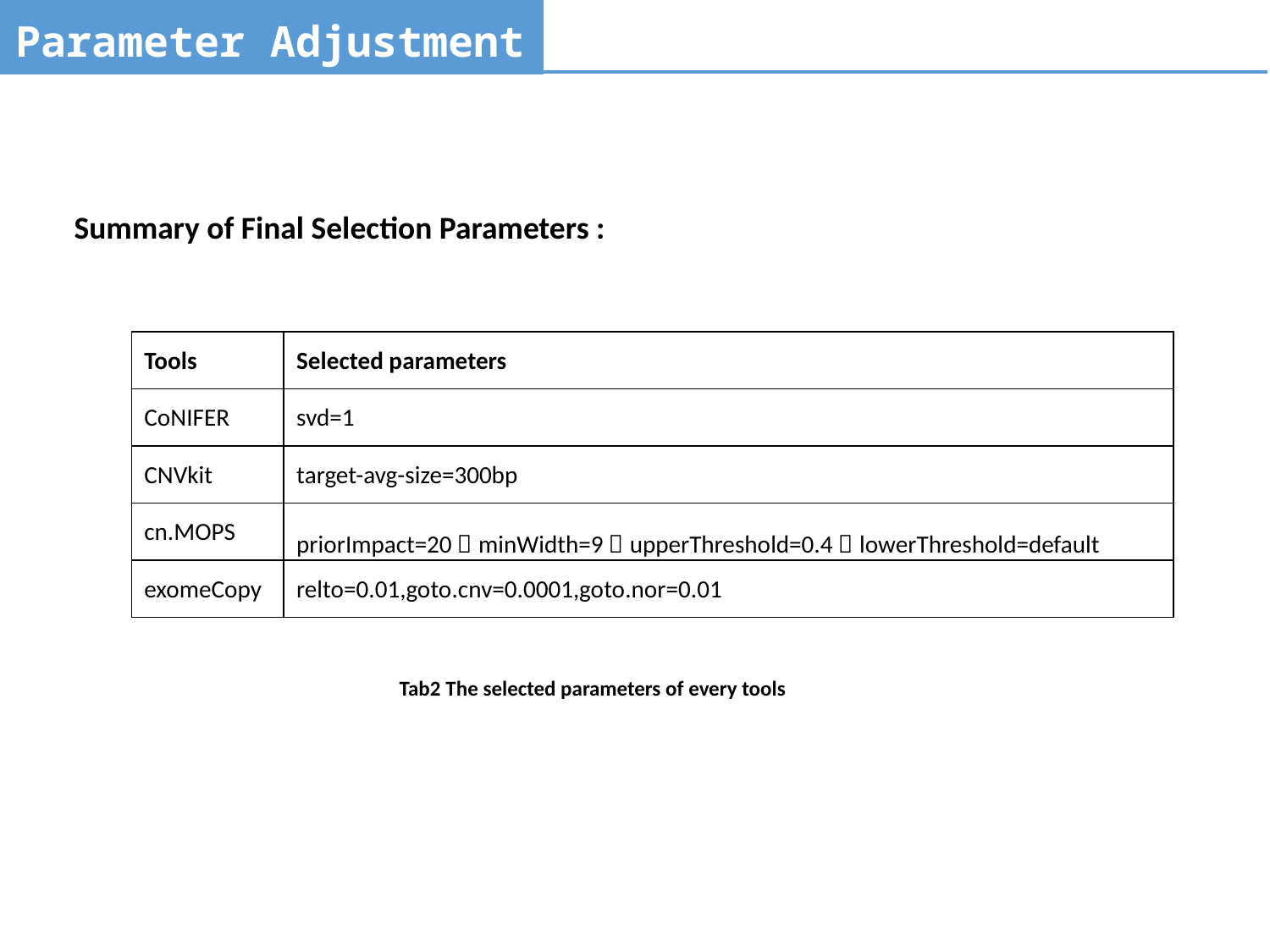

Parameter Adjustment
Summary of Final Selection Parameters :
| Tools | Selected parameters |
| --- | --- |
| CoNIFER | svd=1 |
| CNVkit | target-avg-size=300bp |
| cn.MOPS | priorImpact=20，minWidth=9，upperThreshold=0.4，lowerThreshold=default |
| exomeCopy | relto=0.01,goto.cnv=0.0001,goto.nor=0.01 |
 Tab2 The selected parameters of every tools
